# Impulse oscillometry-derived respiratory mechanical abnormalities and fibrotic disease progression in interstitial lung disease: a single-center cohort study

**DOI:** 10.3389/fphys.2026.1962001

**Published:** 2026-10-19

**Authors:** Chuhan Zhang, Yiying Wang, Zhen Feng, Yitian Sun, Maowei Zhang, Yanan Liu, Bi Chen

**Affiliations:** Department of Respiratory and Critical Care Medicine, the Affiliated Hospital of Xuzhou Medical University, Xuzhou, China

**Keywords:** fibrotic disease progression, impulse oscillometry, interstitial lung disease, pulmonary fibrosis, respiratory impedance, respiratory mechanics

## Abstract

**Objective:**

To examine whether continuous impulse oscillometry (IOS)-derived respiratory mechanical measures are associated with subsequent fibrotic disease progression in interstitial lung disease (ILD).

**Methods:**

In this single-center ambispective cohort study, 255 adults with fibrotic ILD underwent baseline spirometry, diffusing capacity testing and IOS. Fibrotic disease progression was defined using a PPF-like composite requiring at least two of three domains (worsening respiratory symptoms, physiological progression and radiological progression) within a 9–15-month assessment window. All IOS parameters were analyzed continuously using Firth penalized logistic regression, adjusted for age, sex, height and baseline FVC % predicted. X5 was additionally assessed using continuous scaling and restricted cubic splines. ROC analyses, non-IPF sensitivity analyses, and bootstrap validation were exploratory.

**Results:**

Fibrotic disease progression occurred in 41 of 255 patients (16.1%). A 1-SD worsening in X5 was associated with higher odds of progression (OR 8.89, 95% CI 4.66–19.47; P<0.001), as were AX (OR 1.67, 95% CI 1.22–2.38; P = 0.001) and Fres (OR 1.61, 95% CI 1.11–2.36; P = 0.012). Each 0.01 kPa·s/L increase in X5 was associated with lower odds of progression (adjusted OR 0.67, 95% CI 0.59–0.76; P<0.001), and the X5–progression association was nonlinear (P for nonlinearity<0.001). After exclusion of IPF, X5 and AX remained associated with progression. Bootstrap validation showed improved discrimination after adding AX or X5, but did not constitute external validation.

**Conclusions:**

Continuous IOS measures, particularly X5 and AX, were associated with subsequent fibrotic disease progression in this single-center ILD cohort, with a weaker signal for Fres. The X5 association was nonlinear and remained evident after exclusion of IPF. ROC-derived thresholds were unstable and should be considered exploratory. These findings support further evaluation of oscillometric respiratory-mechanical abnormalities in larger externally validated cohorts but do not establish clinically applicable predictive thresholds.

## 1 Introduction

Interstitial lung disease (ILD) encompasses a heterogeneous group of diffuse parenchymal lung disorders that may lead to worsening symptoms, a decline in lung function and premature mortality (Pritchard et al., 2019). The term progressive pulmonary fibrosis (PPF) has been proposed to describe patients with non-idiopathic pulmonary fibrosis (non-IPF) ILD, and international guidance recognizes progression using clinical, physiological and radiological domains (Cottin et al., 2018; George et al., 2020; Raghu et al., 2022; Rajan et al., 2023; Chen et al., 2023; Naqvi et al., 2024). Because the present cohort included both IPF and non-IPF ILD patients, we used the term “fibrotic disease progression” to describe a PPF-like composite outcome adapted from established progression criteria.

Although ILD is traditionally considered a parenchymal disorder, peripheral airway and lung mechanical abnormalities may contribute to disease expression and progression. Such abnormalities may reflect peribronchiolar fibrosis, airway inflammation, parenchymal remodeling, reduced compliance and heterogeneous regional time constants (Xiao et al., 2020; Panagopoulos et al., 2022; Barkas et al., 2023; Xu et al., 2023). In patients with fibrotic ILD, impulse oscillometry (IOS) parameters such as X5 and AX are influenced not only by small airway caliber but also by parenchymal stiffness, elastic recoil and peripheral mechanical load. Therefore, abnormal IOS parameters should be interpreted as markers of peripheral respiratory mechanical impairment rather than direct evidence of isolated small airway disease.

IOS is used to assess respiratory system resistance and reactance during tidal breathing and may provide complementary information to that obtained by conventional spirometry (King et al., 2020; Porojan-Suppini et al., 2020; Mou et al., 2024). Previous studies have described cross-sectional associations between IOS parameters and ILD severity (Soave et al., 2020; Gogali et al., 2024; Kotsiou et al., 2026), and emerging longitudinal evidence suggests that oscillometry may help identify ILD progression (Dube et al., 2026); however, the prognostic value of IOS parameters remains insufficiently established. We therefore examined the associations of continuous IOS measures with subsequent fibrotic disease progression as the principal analysis. As secondary aims, we evaluated correlations between IOS and spirometric indices, explored the discriminative overlap of IOS with a spirometry-defined small-airway impairment comparator, assessed the nonlinear relationship between X5 and progression, and examined the robustness of the main findings in non-IPF ILD and in internally validated exploratory models.

## 2 Materials and methods

### 2.1 Study design and participants

This was a single-center ambispective observational cohort study conducted at the Department of Respiratory and Critical Care Medicine, The Affiliated Hospital of Xuzhou Medical University. The ambispective design incorporated retrospectively available routine clinical, HRCT, pulmonary function and IOS data from patients evaluated between September 2023 and May 2025, together with prospective longitudinal follow-up after formal study enrollment. Formal enrollment commenced after ethics approval on December 5, 2023, and written informed consent was obtained before enrollment. A total of 348 patients with suspected ILD were screened. Thirty-eight were excluded at the initial screening stage (age <18 years, n=2; COPD, asthma, clinically dominant bronchiectasis or other airway-dominant disease, n=21; acute severe illness, n=5; malignancy and/or radiotherapy/chemotherapy, n=4; incomplete baseline clinical records, n=6), leaving 310 potentially eligible patients. A further 55 were excluded because baseline HRCT showed no definite evidence of pulmonary fibrosis and/or key baseline IOS/PFT data were unavailable, yielding a final fibrotic ILD cohort of 255 patients. ILD diagnosis was established by multidisciplinary discussion based on clinical features, exposure history, HRCT, serological findings and histopathology when available. Baseline was defined as the date of the first acceptable IOS assessment performed at or after ILD diagnosis, which could precede formal study enrollment when the assessment had been obtained as part of routine clinical care. After enrollment, participants underwent longitudinal follow-up for outcome assessment. Participant flow is summarized in **Figure 1**.

**Figure 1:**
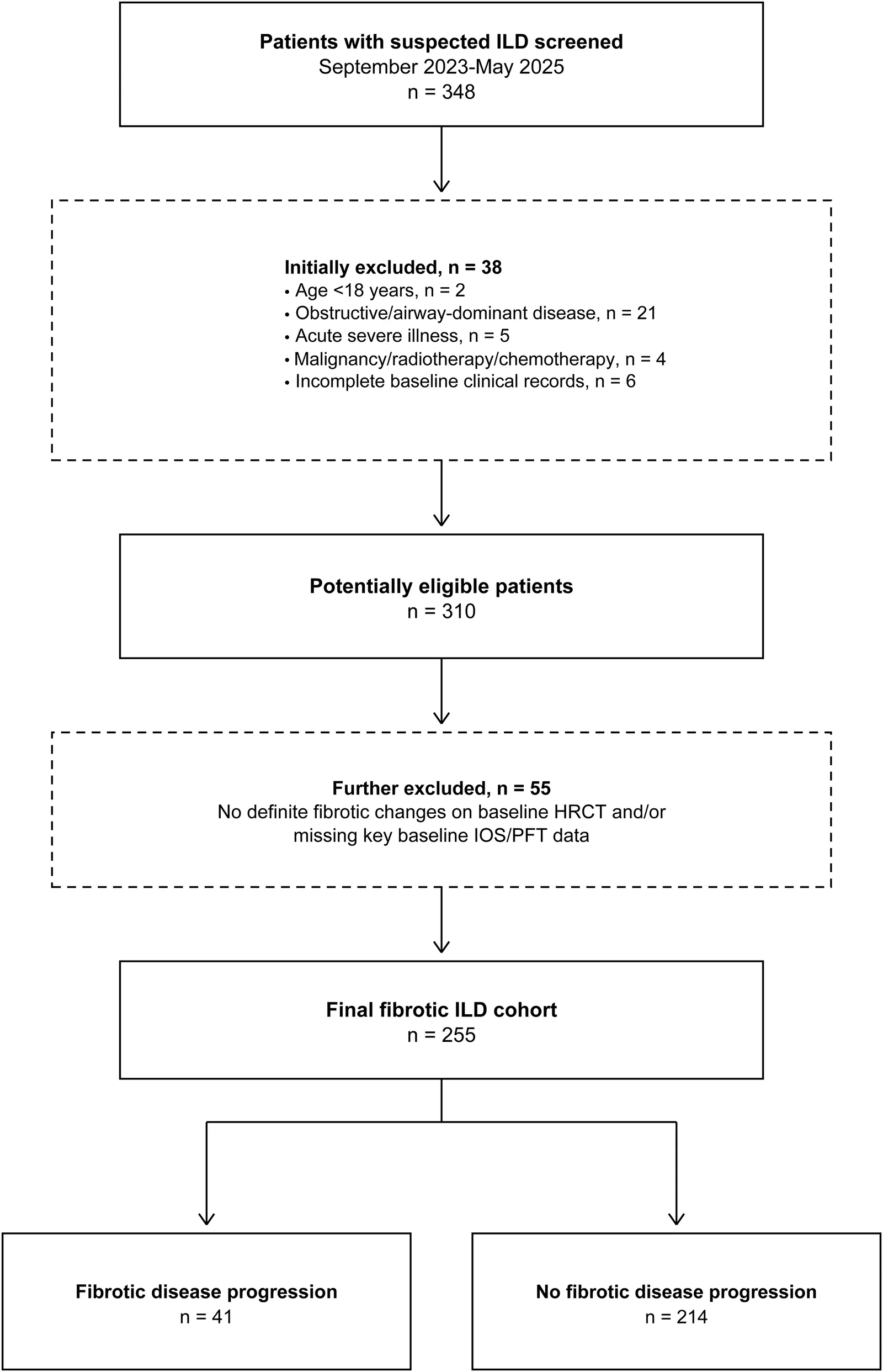
Participant flow diagram for the fibrotic ILD cohort. Of 348 screened patients, 38 were excluded at initial screening and a further 55 were excluded because baseline HRCT lacked definite fibrotic changes and/or key baseline IOS/PFT data were unavailable, yielding 255 patients in the final cohort.

Healthy controls were recruited from individuals undergoing routine health examinations at the same institution and were used only as a descriptive physiological reference group. The study was approved by the Ethics Committee of The Affiliated Hospital of Xuzhou Medical University on December 5, 2023 (approval No. XYFY2023-KL430-01). Formal study enrollment commenced thereafter, and written informed consent was obtained from all participants before enrollment. Eligibility for the ILD cohort required age ≥18 years, MDD-confirmed fibrotic ILD with pulmonary fibrosis on baseline HRCT, complete baseline clinical data, acceptable-quality spirometry and IOS, and follow-up information sufficient for progression adjudication. The healthy-control comparison is reported in the **Supplementary Material** and was not used as primary inferential evidence. No additional patients were excluded solely because of loss to follow-up, death, lung transplantation, or insufficient follow-up information during the prespecified outcome-assessment window.

### 2.2 ILD classification

ILD subtypes were classified as IPF, connective tissue disease-associated ILD (CTD-ILD), or other ILDs, including hypersensitivity pneumonitis, idiopathic nonspecific interstitial pneumonia, organizing pneumonia-predominant ILD and unclassifiable ILD according to international recommendations and MDD conclusions. Subtypes were recorded for descriptive analyses; primary analyses were not stratified by subtype because the study focused on respiratory mechanical phenotypes associated with progression.

### 2.3 Pulmonary function testing

Spirometry was performed using a MasterScreen PFT system (Jaeger, Germany) according to ATS/ERS standards (Graham et al., 2019; Ahookhosh et al., 2023). The predicted values were derived from population-appropriate reference equations. Spirometry-defined small airway impairment (SAI) was defined as at least two of three forced expiratory flows, FEF25–75%, FEF50% and FEF75%, were below 65% of the predicted values (Contoli et al., 2010; Katsoulis et al., 2016; Chen et al., 2024). Spirometry-defined SAI was used only in secondary analyses to characterize the physiological overlap between spirometry and IOS and was not treated as a diagnostic reference standard. Mid-expiratory flows are strongly dependent on lung volume and FVC; therefore, reduced FEF25–75%, FEF50% or FEF75% in restrictive ILD may occur even in the absence of isolated small-airway disease.

IOS was performed using an MS-IOS system (Jaeger, Germany) according to European Respiratory Society technical standards (King et al., 2020) before spirometry. The participants were seated, wore a nose clip and supported their cheeks. At least three technically acceptable recordings during stable tidal breathing were obtained, excluding artifacts, and mean values were used. The analyzed IOS parameters were R5, R20, the device-reported mean R5–R20, X5, Fres and AX. The mean R5–R20 was extracted directly from the IOS system output and was not manually recalculated from the separately exported R5 and R20 summary values. Accordingly, this device-reported parameter was analyzed as a manufacturer-derived summary measure and was not interpreted as the arithmetic difference between the separately reported R5 and R20 values. R5, R20, mean R5–R20 and X5 were expressed as kPa·s/L, Fres as Hz and AX according to the device output unit. More negative X5 values indicated greater abnormalities.

### 2.4 Follow-up and outcome assessment

Progression status was evaluated using information accrued within a prespecified 9–15-month window around the 12-month follow-up point. The primary outcome was fibrotic disease progression, defined using a PPF-like framework adapted for a cohort including both IPF and non-IPF ILD. Progression required at least two of three domains in the absence of an alternative explanation: (1) worsening respiratory symptoms, defined as documented new or increased exertional dyspnea and/or cough considered attributable to ILD, in the absence of an evident alternative acute explanation; (2) physiological progression, defined as a ≥5% absolute decline in FVC % predicted and/or a ≥10% absolute decline in DLCO % predicted; and (3) radiological progression on HRCT, including increased traction bronchiectasis/bronchiolectasis, reticulation, honeycombing, architectural distortion and/or lung-volume loss. Domain availability was recorded separately, and an unavailable domain was not treated as a negative finding. Outcomes were independently assessed by two ILD specialists blinded to baseline IOS results, with consensus resolution and third-reviewer involvement when needed; radiological progression was assessed by a thoracic radiologist blinded to IOS findings. For patients meeting progression criteria, the outcome-assessment date was defined as the date on which the second positive domain became documented. For patients without progression, the assessment date was defined as the latest date within the prespecified window at which sufficient available information established that the patient did not meet at least two progression domains. For non-progressors, sufficient information required that the available domain pattern precluded fulfillment of the prespecified two-of-three progression criterion; unavailable domains were never assumed to be negative.

### 2.5 Statistical analysis

Continuous variables are summarized as the mean ± SD or median (IQR), and categorical variables are summarized as counts and percentages. For between-group comparisons, t tests or Wilcoxon rank-sum tests were used for continuous variables, and chi-square or Fisher’s exact tests were used for categorical variables. The analyses were organized into primary, secondary, and exploratory components. The primary analysis focused on the associations between continuous IOS measurements and fibrotic disease progression, whereas subsequent analyses evaluated the robustness, discrimination, and potential clinical interpretation of the findings. Because the healthy controls were not age matched to the ILD cohort, between-group comparisons were interpreted as descriptive reference comparisons rather than primary inferential analyses. Within the ILD cohort, correlations between IOS and pulmonary function parameters were assessed using Spearman correlation coefficients.

ROC analysis against spirometry-defined SAI was retained as a secondary exploratory analysis to quantify physiological overlap rather than to establish clinical diagnostic thresholds. Optimal cutoffs were selected using the Youden index. AUC, sensitivity, specificity, positive predictive value, negative predictive value and accuracy were calculated. Apparent AUCs and their 95% confidence intervals were estimated using DeLong’s method, whereas exact binomial 95% confidence intervals were calculated for classification measures. Internal validation of the secondary ROC analyses used 500 ordinary bootstrap resamples. In each bootstrap resample, ROC analysis was repeated using the continuous IOS measure, and the optimal cutoff was re-estimated using the Youden index. The continuous IOS measure was evaluated in the corresponding out-of-bag sample for AUC, whereas the bootstrap-derived cutoff was applied to the out-of-bag sample to assess classification performance. The.632+ bootstrap method was additionally used to obtain optimism-corrected AUC estimates by combining apparent and out-of-bag performance. Repeated 10-fold cross-validation with 10 repetitions was retained as a secondary assessment of AUC reproducibility. Because the cutoffs were internally optimized against a spirometric comparator and varied across bootstrap samples, all cutoff-based analyses were considered exploratory.

The primary association analysis treated all six IOS parameters as continuous variables. Each IOS parameter was analyzed separately using Firth penalized logistic regression, with effect estimates expressed per 1-SD worsening. Firth penalized logistic regression was used for all IOS association models because of the limited number of progression events and the occurrence of separation for X5. A deliberately parsimonious clinically selected adjustment set included age, sex, height and baseline FVC % predicted. Systemic corticosteroid, antifibrotic and immunosuppressive therapy were recorded according to treatment status at baseline; post-baseline treatment changes were not modeled. Treatment variables were not included in the principal adjusted model to reduce model complexity and concern for confounding by indication. Benjamini–Hochberg false-discovery-rate-adjusted P values were calculated across the six continuous IOS analyses as a multiplicity sensitivity analysis.

X5 was additionally analyzed per 0.01 kPa·s/L increase, where a higher value represents a less negative and therefore less abnormal reactance. Nonlinearity was assessed using a restricted cubic spline for X5 within the Firth penalized logistic regression framework, with three knots at the 10th, 50th and 90th percentiles. A sensitivity analysis excluded patients with IPF and repeated the reduced Firth models using the full-cohort SD scaling. Exploratory discrimination and incremental model-performance analyses were performed to assess the potential added value of IOS measures rather than to establish a clinically deployable prediction model. A clinical base model containing age, sex, height and baseline FVC % predicted was compared with models additionally containing AX, linear X5 or spline-modeled X5. These exploratory model-performance analyses were based on standard unpenalized logistic regression and were fitted separately from the principal Firth penalized association models. Internal validation used 500 bootstrap resamples of the complete modeling process. Optimism-corrected AUC, Brier score, calibration intercept and calibration slope were reported. Uncertainty in incremental model performance was assessed using a nested bootstrap procedure. Five hundred outer bootstrap samples were drawn from the original cohort. Within each outer sample, optimism correction of model performance was performed using 200 inner bootstrap resamples of the complete modeling process. For the spline-modeled X5 model, percentile-based knot locations were re-estimated within each training resample. Corrected differences in AUC and Brier score between each IOS-augmented model and the clinical base model were calculated within each outer sample, and percentile-based 95% confidence intervals were obtained from the outer-bootstrap distributions. These analyses were exploratory and were not interpreted as external validation of a clinical prediction model. To assess the robustness of the findings to variation in follow-up timing, a sensitivity analysis was performed after restricting the cohort to participants whose actual outcome-assessment dates fell within 10–14 months after baseline, using the same Firth penalized logistic models, covariate adjustment and full-cohort 1-SD scaling as in the primary 9–15-month analysis.

## 3 Results

### 3.1 Study population and baseline characteristics

Of 348 screened patients with suspected ILD, 38 were excluded at initial screening and a further 55 were excluded because of absent definite baseline fibrotic HRCT findings and/or missing key baseline IOS/PFT data; 255 patients with fibrotic ILD comprised the final cohort (**Figure 1**). Fibrotic disease progression occurred in 41 patients (16.1%), and 214 patients did not meet progression criteria. Baseline characteristics according to progression status are shown in **Table 1**. Sex distribution, ILD subtype and baseline use of systemic corticosteroids, antifibrotic therapy and immunosuppressive therapy did not differ materially between groups. Progressors had more abnormal Fres, X5 and AX values at baseline, whereas between-group differences for R20 and device-reported Mean R5–R20 were small. Healthy-control data are retained only as a descriptive reference comparison in the **Supplementary Material**.

**Table 1:** Baseline characteristics according to fibrotic disease progression status.

Variable	Progression (n=41)	No progression (n=214)	P value
Demographics			
Age, years	64.1 ± 12.2	65.7 ± 11.5	0.450
Male sex, n (%)	21 (51.2%)	114 (53.3%)	0.809
Height, cm	160.2 ± 8.3	161.8 ± 8.8	0.254
BMI, kg/m²	24.3 ± 3.6	24.8 ± 3.4	0.372
ILD subtype, n (%)			0.937
IPF	13 (31.7%)	74 (34.6%)	
CTD-ILD	13 (31.7%)	66 (30.8%)	
Other ILD	15 (36.6%)	74 (34.6%)	
Baseline treatment, n (%)			
Systemic corticosteroid use	24 (58.5%)	138 (64.5%)	0.468
Antifibrotic therapy	17 (41.5%)	78 (36.4%)	0.543
Immunosuppressive therapy	7 (17.1%)	49 (22.9%)	0.409
Pulmonary function			
FVC, % predicted	77.4 ± 19.5	83.5 ± 20.9	0.078
DLCO, % predicted	59.7 ± 18.4	61.9 ± 18.2	0.494
Impulse oscillometry			
Fres, Hz	16.96 (14.89–20.61)	15.39 (12.41–17.89)	0.002
R5, kPa·s/L	0.43 (0.33–0.56)	0.37 (0.31–0.49)	0.037
R20, kPa·s/L	0.30 (0.23–0.38)	0.29 (0.23–0.35)	0.712
Device-reported Mean R5–R20, kPa·s/L	0.36 (0.28–0.48)	0.33 (0.27–0.42)	0.111
X5, kPa·s/L	−0.11 (−0.14 to −0.10)	−0.05 (−0.07 to −0.02)	<0.001
AX, kPa/L	0.83 (0.49–1.26)	0.34 (0.17–0.64)	<0.001

Continuous variables are presented as mean ± SD or median (IQR), as appropriate; categorical variables as n (%). The P value for ILD subtype represents the overall 3-category comparison. Baseline treatment variables indicate treatment status at baseline. Mean R5–R20 is the subject-level device-reported parameter exported directly by the IOS system.

### 3.2 IOS parameters, spirometry-defined impairment and ROC-derived thresholds

Within the ILD cohort, IOS parameters were weakly to moderately correlated with spirometric small-airway indices (**Figure 2**). The strongest correlation was observed between FEF75% predicted and AX (ρ=−0.451; P<0.001), followed by FEF75% predicted and Fres (ρ=−0.396). Using the prespecified exploratory spirometric criterion, 68 patients (26.7%) were classified as having SAI and 187 (73.3%) as not having SAI. Secondary ROC analyses demonstrated modest-to-moderate discriminative overlap between IOS measures and spirometry-defined SAI, with AUCs ranging from 0.620 to 0.693. AX had the highest AUC (0.693, 95% CI 0.620–0.766), followed by X5 (0.676, 95% CI 0.599–0.753). However, the internally derived cutoff values varied across bootstrap samples, and these thresholds were therefore not treated as clinically validated cutoffs. Detailed ROC classification performance is presented in **Supplementary Table S7** and the corresponding ROC curves are shown in **Supplementary Figure S2**. Bootstrap/OOB,.632+-corrected and repeated 10-fold cross-validation AUC estimates were broadly similar to the apparent AUCs, whereas the internally derived cutoff values showed substantial variability across bootstrap samples, particularly for X5 (−0.130 to −0.050 kPa·s/L) and AX (0.188–1.241 kPa/L) (**Supplementary Table S8**).

**Figure 2:**
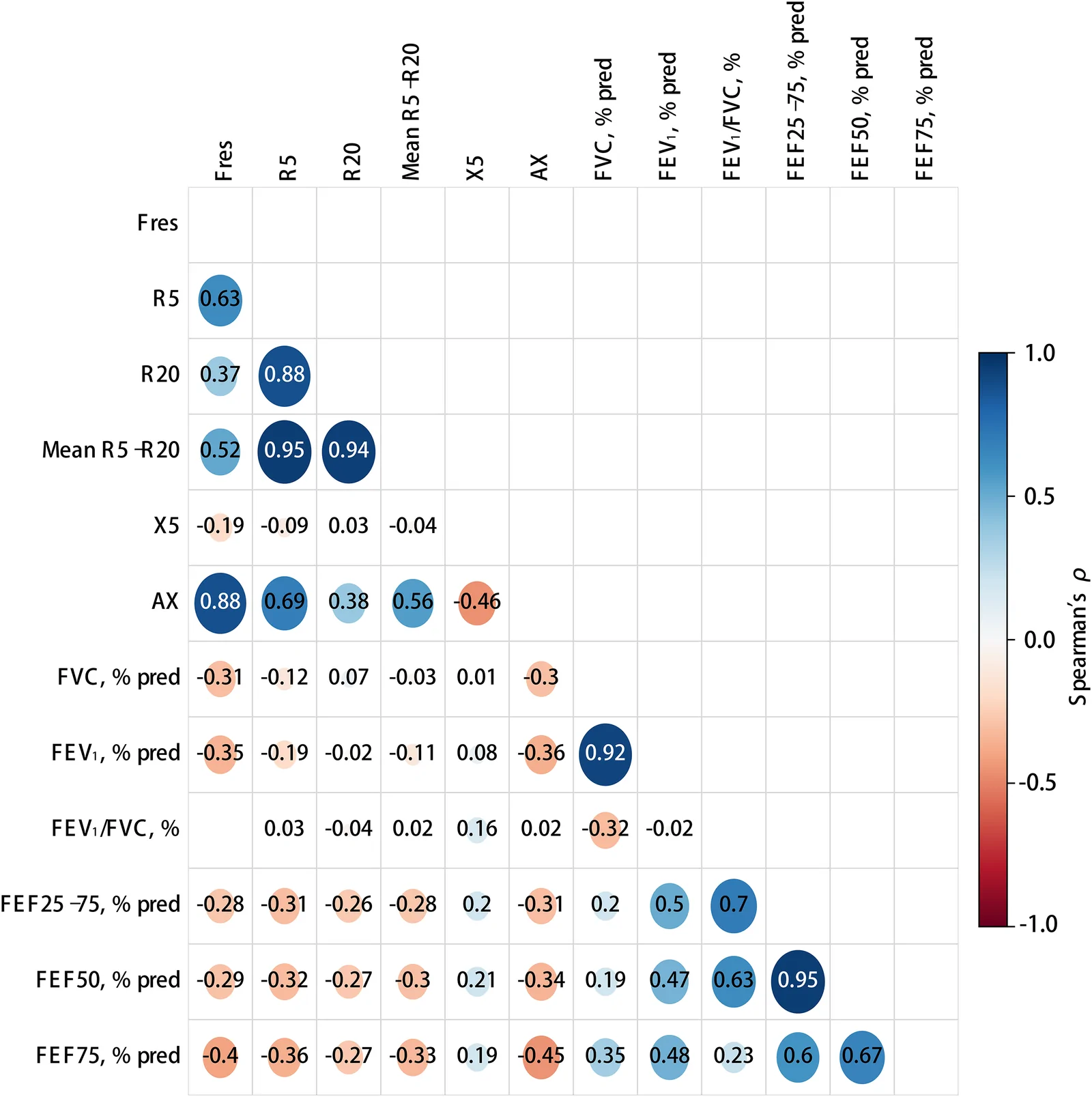
Spearman correlation matrix of impulse oscillometry and pulmonary function parameters. Circle size and color intensity represent the magnitude and direction of Spearman’s correlation coefficient (rho), respectively.

### 3.3 Associations of IOS measures with fibrotic disease progression

During follow-up, fibrotic disease progression occurred in 41 of 255 patients (16.1%). Among progressors, the symptom domain was evaluable in 30/41 (73.2%), the physiological domain in 39/41 (95.1%) and follow-up HRCT in 29/41 (70.7%). Among evaluable progressors, symptom worsening occurred in 24/30 (80.0%), physiological progression in 36/39 (92.3%) and radiological progression in 23/29 (79.3%). The mutually exclusive documented progression-domain combinations were symptoms plus physiology without documented radiological progression in 18 patients (43.9%), symptoms plus radiology without documented physiological progression in 5 (12.2%), physiology plus radiology without documented symptom worsening in 17 (41.5%), and all three documented domains in 1 (2.4%). Domain availability and component frequencies in progressors and non-progressors are summarized in the **Supplementary Material**.

In the revised principal analysis, continuous IOS measures were evaluated using Firth penalized logistic regression. After adjustment for age, sex, height and baseline FVC % predicted, a 1-SD worsening in X5 was associated with substantially higher odds of fibrotic disease progression (OR 8.89, 95% CI 4.66–19.47; P<0.001). AX (OR 1.67, 95% CI 1.22–2.38; P = 0.001) and Fres (OR 1.61, 95% CI 1.11–2.36; P = 0.012) were also associated with progression. The associations for R5, R20 and device-reported Mean R5–R20 were not statistically robust after reduced adjustment and multiplicity sensitivity analysis (**Table 2**; **Figure 3**). Benjamini–Hochberg FDR-adjusted P values were <0.001 for X5, 0.004 for AX and 0.024 for Fres.

**Table 2:** Associations of continuous IOS parameters with fibrotic disease progression.

IOS parameter	1-SD	Crude OR (95% CI)	P value	Adjusted OR (95% CI)	P value	FDR-adjusted P
Fres	4.801	1.59 (1.16–2.21)	0.004	1.61 (1.11–2.36)	0.012	0.024
R5	0.163	1.38 (1.03–1.85)	0.032	1.36 (0.96–1.96)	0.085	0.127
R20	0.094	1.17 (0.84–1.59)	0.337	1.11 (0.75–1.60)	0.587	0.587
Mean R5–R20	0.126	1.34 (0.99–1.81)	0.057	1.33 (0.92–1.90)	0.125	0.150
X5	0.055	8.04 (4.47–16.11)	<0.001	8.89 (4.66–19.47)	<0.001	<0.001
AX	0.775	1.60 (1.22–2.18)	<0.001	1.67 (1.22–2.38)	0.001	0.004

Odds ratios were estimated using Firth penalized logistic regression and are expressed per 1-SD worsening. The adjusted model included age, sex, height and baseline FVC % predicted. For X5, worsening indicates a more negative value; for Fres, R5, R20, device-reported Mean R5–R20 and AX, worsening indicates a higher value. FDR-adjusted P values were calculated using the Benjamini–Hochberg procedure across the six IOS analyses.

**Figure 3:**
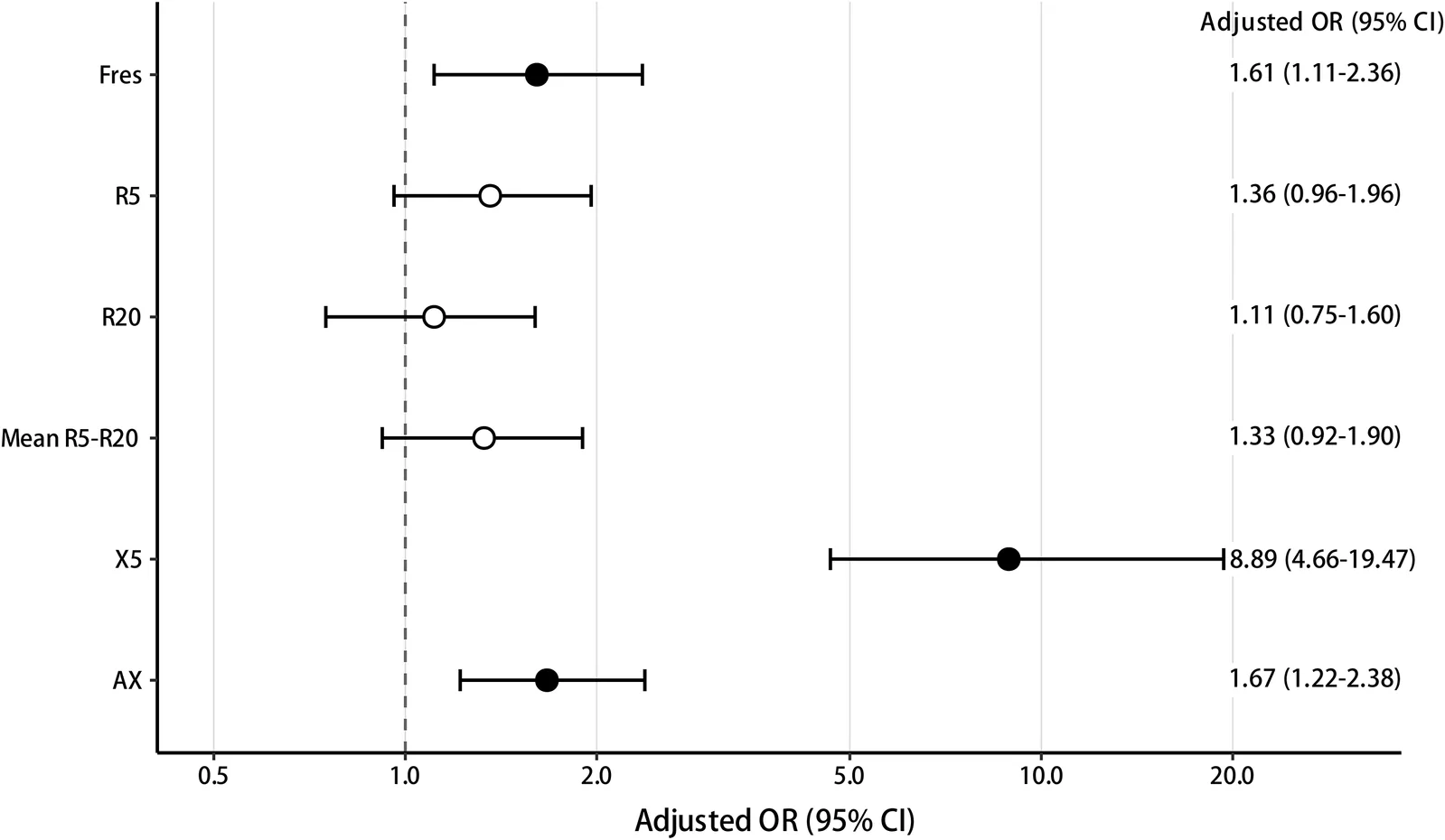
Forest plot of adjusted odds ratios for fibrotic disease progression per 1-SD worsening in each continuous IOS parameter. Estimates were obtained from Firth penalized logistic regression adjusted for age, sex, height and baseline FVC % predicted. For X5, worsening denotes a more negative value; for the other IOS parameters, worsening denotes a higher value. Filled circles indicate P<0.05, whereas open circles indicate P≥0.05.

For X5, each 0.01 kPa·s/L increase (a less negative value) was associated with lower odds of progression (adjusted OR 0.67, 95% CI 0.59–0.76; P<0.001). Restricted cubic spline analysis demonstrated evidence of nonlinearity (P overall<0.001; P for nonlinearity<0.001; **Supplementary Figure S1**), indicating that a single linear effect estimate does not fully describe the X5–progression relationship. Using the internally derived X5 cutoff, 41 progression events occurred among 76 participants with abnormal X5 and no events occurred among 179 participants with normal X5. Sensitivity was 100.0% (exact 95% CI 91.4–100.0%), specificity 83.6% (78.0–88.3%), PPV 53.9% (42.1–65.5%) and NPV 100.0% (98.0–100.0%). This complete-separation pattern was interpreted as cohort-specific and exploratory rather than as evidence of zero risk or a validated clinical rule-out threshold.

In the sensitivity analysis excluding patients with IPF (n=168; 28 progression events), X5 remained strongly associated with progression (adjusted OR per 1-SD worsening 8.55, 95% CI 4.03–22.53; P<0.001), and AX remained associated with progression (OR 1.47, 95% CI 1.05–2.16; P = 0.027). In exploratory model-performance analyses, the optimism-corrected AUC was 0.585 for the clinical base model, 0.703 after adding AX, 0.925 after adding linear X5 and 0.921 after adding spline-modeled X5. Compared with the clinical base model, the optimism-corrected ΔAUC was +0.119 (95% CI + 0.020 to +0.215) after addition of AX, + 0.340 (+0.222 to +0.419) after addition of linear X5, and +0.336 (+0.241 to +0.428) with spline-modeled X5. Corresponding Brier-score improvements were +0.002 (95% CI −0.002 to +0.022), +0.043 (+0.018 to +0.081), and +0.055 (+0.036 to +0.089), respectively (**Table 3**). These model comparisons were internally validated but remain exploratory and should not be interpreted as external clinical prediction validation. The median actual outcome-assessment interval was 12.1 months (IQR 11.4–12.9) in progressors and 12.0 months (IQR 11.3–12.8) in non-progressors. Overall, 227 of 255 participants (89.0%) had an assessment date within 10–14 months after baseline (**Supplementary Table S10**). In the 10–14-month sensitivity analysis, the main findings were directionally consistent with the primary analysis: X5 remained strongly associated with progression (adjusted OR per 1-SD worsening 7.65, 95% CI 3.72–17.20; P<0.001), and AX also remained associated with progression (OR 1.55, 95% CI 1.07–2.27; P = 0.021), whereas Fres, R5, R20 and device-reported Mean R5–R20 were not statistically significant (**Supplementary Table S11**).

**Table 3:** Exploratory internally validated model performance for fibrotic disease progression.

Model	Optimism-corrected AUC	Optimism-corrected brier score	Optimism-corrected calibration intercept	Optimism-corrected calibration slope	Corrected ΔAUC vs base (95% CI)	Brier improvement vs base (95% CI)
Clinical base model	0.585	0.137	−0.383	0.773	—	—
Base + AX	0.703	0.134	−0.318	0.811	+0.119 (+0.020 to +0.215)	+0.002 (−0.002 to +0.022)
Base + linear X5	0.925	0.094	−0.061	0.952	+0.340 (+0.222 to +0.419)	+0.043 (+0.018 to +0.081)
Base + X5 RCS	0.921	0.081	−0.007	0.871	+0.336 (+0.241 to +0.428)	+0.055 (+0.036 to +0.089)

The clinical base model included age, sex, height and baseline FVC % predicted. Apparent model performance was internally validated using 500 bootstrap resamples of the complete modeling process. Uncertainty in incremental model performance was quantified using a nested bootstrap procedure with 500 outer bootstrap resamples; within each outer sample, optimism correction was performed using 200 inner bootstrap resamples. For the X5 RCS model, spline knot locations were re-estimated within each training resample. Corrected differences in AUC and Brier score between each IOS-augmented model and the clinical base model were calculated within each outer bootstrap sample, and percentile-based 95% confidence intervals were derived from the outer-bootstrap distribution. Lower Brier scores indicate better overall prediction accuracy; ideal calibration intercept and slope are 0 and 1, respectively. These analyses are exploratory and do not constitute external validation. Brier improvement was calculated as the optimism-corrected Brier score of the clinical base model minus that of the IOS-augmented model; therefore, positive values indicate improvement. Differences were calculated from unrounded estimates.

## 4 Discussion

The revised analysis indicates that continuous oscillometric abnormalities, particularly X5 and AX, are associated with subsequent fibrotic disease progression in ILD, with a weaker signal for Fres. In contrast, R5, R20 and device-reported Mean R5–R20 were not statistically robust after parsimonious adjustment and multiplicity sensitivity analysis. This pattern shifts the emphasis away from dichotomized IOS thresholds and toward continuous reactance-related measures that may reflect the combined mechanical effects of parenchymal fibrosis, reduced compliance and peripheral heterogeneity.

The strong X5 association should nevertheless be interpreted cautiously. A 0.01 kPa·s/L increase in X5, corresponding to a less negative and less abnormal value, was associated with lower odds of progression, and restricted cubic spline analysis showed significant nonlinearity. Thus, the relationship cannot be adequately summarized by a single constant linear effect across the full X5 range. The complete-separation pattern observed with the internally derived binary X5 cutoff is therefore best viewed as a cohort-specific exploratory finding rather than as a clinically validated rule-out threshold.

AX showed a more modest but consistent association with progression. Exploratory bootstrap validation suggested that adding AX improved optimism-corrected discrimination relative to the clinical base model, although the improvement in Brier score was small and uncertain. Adding X5 produced a substantially larger improvement in discrimination and overall prediction error. These results address incremental model performance at an exploratory level, but the limited number of progression events and the absence of external validation preclude claims of established clinical prediction or utility.

The findings remained broadly consistent after exclusion of patients with IPF, with X5 and AX retaining associations with progression. This is relevant because guideline-defined PPF formally applies to non-IPF fibrotic ILD, whereas the present mixed cohort used a PPF-like fibrotic disease progression endpoint. Healthy-control comparisons should likewise remain secondary because the controls were substantially younger than the ILD cohort and absolute oscillometric values are influenced by demographic and anthropometric factors.

Several limitations should be acknowledged. This was a single-center ambispective cohort with only 41 progression events, and all multivariable and model-performance analyses should therefore be considered exploratory. The spirometric SAI definition was an imperfect physiological comparator rather than a diagnostic gold standard, particularly because mid-expiratory flows depend on lung volume and FVC in restrictive disease. ROC-derived thresholds were internally optimized and unstable across resamples. Follow-up HRCT was not uniformly available and was more frequently available among progressors than non-progressors, which may have introduced ascertainment bias. Treatment changes during follow-up were not modeled, and treatment variables were omitted from the principal adjusted model because of limited events and concern for confounding by indication. External validation in larger cohorts with standardized outcome-assessment timing is required.

## 5 Conclusions

Continuous IOS measures, particularly X5 and AX, were associated with subsequent fibrotic disease progression in this single-center ILD cohort, with a weaker signal for Fres. The association between X5 and progression was nonlinear and remained evident after exclusion of patients with IPF. ROC-derived thresholds were unstable and should be considered exploratory. These findings support further evaluation of oscillometric respiratory-mechanical abnormalities in larger externally validated cohorts but do not establish clinically applicable predictive thresholds.

## Data Availability

The datasets presented in this article are not readily available because of patient confidentiality restrictions imposed by the Ethics Committee. Requests to access the datasets should be directed to Bi Chen (chenbi207@126.com).

## Supplementary material

Supplementary Table 1 Availability and positivity of progression domains.

Supplementary Table 2 Mutually exclusive progression-component combinations among progressors.

Supplementary Table 3 Continuous X5 analysis per 0.01 kPa·s/L increase. A + 0.01 kPa·s/L change represents a less negative and therefore less abnormal X5 value. The adjusted model included age, sex, height and baseline FVC % predicted.

Supplementary Table 4 Exploratory X5 cutoff classification for fibrotic disease progression. The threshold was internally derived against the spirometry-defined SAI comparator and is presented only as an exploratory cohort-specific threshold. No progression events were observed among participants with normal X5 in this cohort; this should not be interpreted as zero risk or as a validated rule-out threshold.

Supplementary Table 5 Sensitivity analysis after exclusion of patients with IPF (n=168; 28 progression events). Panel **(A)**. Continuous IOS associations in patients with non-IPF ILD. Adjusted odds ratios were estimated using Firth penalized logistic regression adjusted for age, sex, height and baseline FVC % predicted. Effects are expressed per 1-SD worsening using SDs derived from the full cohort. For X5, worsening indicates a more negative value; for all other IOS measures, worsening indicates a higher value. Panel **(B)**. Exploratory classification according to the X5 cutoff in patients with non-IPF ILD. The X5 threshold was derived in the full cohort against spirometry-defined SAI and was applied unchanged in the non-IPF subgroup. Exact binomial 95% confidence intervals are reported for classification measures. No progression events were observed among participants with normal X5 in this cohort; this finding should not be interpreted as evidence of zero risk or as a validated clinical rule-out threshold.

Supplementary Table 6 Secondary exploratory cutoff-based associations with progression. Firth penalized logistic regression was adjusted for age, sex, height and baseline FVC % predicted. Cutoffs were internally derived against spirometry-defined SAI and are presented only as secondary exploratory thresholds.

Supplementary Table 7 Exploratory classification performance of IOS cutoffs against spirometry-defined SAI. The positive reference category was spirometry-defined SAI. These thresholds were internally derived and are not proposed as clinical diagnostic cutoffs.

Supplementary Table 8 Internal validation and stability of ROC-derived IOS thresholds against spirometry-defined small airway impairment. Panel **(A)**. Bootstrap-based internal validation of continuous ROC AUC. Panel **(B)**. Repeated cross-validation. Panel **(C)**. Stability of internally selected Youden cutoffs. Spirometry-defined SAI was used as the positive reference category. Apparent AUCs were calculated from the continuous IOS measurements, with 95% confidence intervals estimated using DeLong’s method. Ordinary bootstrap validation used 500 resamples. Within each bootstrap resample, ROC analysis and Youden-index cutoff selection were repeated; continuous IOS measurements were used to evaluate AUC, whereas the bootstrap-selected cutoff was used to assess classification performance in the corresponding out-of-bag observations. The.632+ estimates combined apparent and out-of-bag AUCs using adaptive.632+ weighting. Repeated 10-fold cross-validation was performed with 10 repetitions. Bootstrap cutoff intervals are percentile-based 95% intervals of the re-estimated Youden thresholds. These analyses were secondary and exploratory.

Supplementary Table 9 Baseline demographic characteristics, spirometric indices and impulse oscillometry parameters in healthy controls and patients with interstitial lung disease. P values are unadjusted. Because healthy controls were substantially younger than patients with ILD and were not age matched, these comparisons are presented only as descriptive physiological reference data and were not used as primary inferential evidence. Device-reported Mean R5–R20 was extracted directly from the IOS system output and should not be interpreted as the arithmetic difference between the separately reported R5 and R20 summary values.

Supplementary Table 10 Actual outcome assessment timing.

Supplementary Table 11 Narrower-window sensitivity analysis for continuous IOS associations. Firth penalized logistic regression adjusted for age, sex, height and baseline FVC % predicted. Odds ratios are expressed per 1-SD worsening using the SD scaling derived from the full cohort.

Supplementary Figure 1 Restricted cubic spline analysis of the nonlinear association between X5 and fibrotic disease progression in patients with interstitial lung disease. The solid line represents the adjusted odds ratio and the shaded area represents the 95% confidence interval. The median X5 value was used as the reference (OR = 1). The model was adjusted for age, sex, height and baseline FVC % predicted. The spline was fitted using Firth penalized logistic regression adjusted for age, sex, height and baseline FVC % predicted.

Supplementary Figure 2 Receiver operating characteristic curves showing the discriminative overlap between impulse oscillometry parameters and spirometry-defined small airway impairment. Curves are shown for Fres, R5, R20, device-reported Mean R5–R20, X5 and AX. The diagonal reference line represents an AUC of 0.50.
