# Supplementary material for: Impulse oscillometry-derived respiratory mechanical abnormalities and fibrotic disease progression in interstitial lung disease: a single-center cohort study
Source: Front Physiol. 2026 Oct 19;17:1962001. doi: 10.3389/fphys.2026.1962001 (PMC13724450; doi:10.3389/fphys.2026.1962001)
Supplement: Supplementary file 1 [file SupplementaryFile1.pdf]

Supplementary Table S1. Availability and positivity of progression domains.

Outcome/domain	Progressors (n=41)	Non-progressors (n=214)
**Symptom domain available**	30/41 (73.2%)	182/214 (85.0%)
**Symptom worsening among evaluable patients**	24/30 (80.0%)	1/182 (0.5%)
**Physiological domain available**	39/41 (95.1%)	210/214 (98.1%)
**Physiological progression among evaluable patients**	36/39 (92.3%)	2/210 (1.0%)
**HRCT domain available**	29/41 (70.7%)	108/214 (50.5%)
**Radiological progression among evaluable patients**	23/29 (79.3%)	4/108 (3.7%)

Supplementary Table S2. Mutually exclusive progression-component combinations among progressors.

**Combination among progressors**	**n (%)**
**Symptoms + physiology, without documented radiological progression**	18 (43.9%)
**Symptoms + radiology, without documented physiological progression**	5 (12.2%)
**Physiology + radiology, without documented symptom worsening**	17 (41.5%)
**All three documented domains**	1 (2.4%)
**Total**	**41 (100%)**

Supplementary Table S3. Continuous X5 analysis per 0.01 kPa·s/L increase.

Analysis	OR (95% CI)	P value
**Crude, per +0.01 kPa·s/L**	0.69 (0.61–0.76)	<0.001
**Adjusted, per +0.01 kPa·s/L**	0.67 (0.59–0.76)	<0.001

Note: A +0.01 kPa·s/L change represents a less negative and therefore less abnormal X5 value. The adjusted model included age, sex, height and baseline FVC % predicted.

Supplementary Table S4. Exploratory X5 cutoff classification for fibrotic disease progression.

X5 category	Progression	No progression	Total
**Abnormal (X5 < −0.085)**	41	35	76
**Normal**	0	179	179
**Total**	**41**	**214**	**255**

Metric	Estimate (exact 95% CI)
**Sensitivity**	100.0% (91.4–100.0%)
**Specificity**	83.6% (78.0–88.3%)
**PPV**	53.9% (42.1–65.5%)
**NPV**	100.0% (98.0–100.0%)
**Accuracy**	86.3% (81.4–90.2%)

Note: The threshold was internally derived against the spirometry-defined SAI comparator and is presented only as an exploratory cohort-specific threshold. No progression events were observed among participants with normal X5 in this cohort; this should not be interpreted as zero risk or as a validated rule-out threshold.

Supplementary Table S5. Sensitivity analysis after exclusion of patients with IPF (n=168; 28 progression events).

Panel A. Continuous IOS associations in patients with non-IPF ILD

IOS parameter	Adjusted OR per 1-SD worsening (95% CI)	P value
**Fres**	1.45 (0.92–2.25)	0.105
**R5**	1.27 (0.86–1.90)	0.220
**R20**	1.20 (0.78–1.82)	0.397
**Mean R5–R20**	1.30 (0.86–1.94)	0.211
**X5**	8.55 (4.03–22.53)	<0.001
**AX**	1.47 (1.05–2.16)	0.027

Notes: Adjusted odds ratios were estimated using Firth penalized logistic regression adjusted for age, sex, height and baseline FVC % predicted. Effects are expressed per 1-SD worsening using SDs derived from the full cohort. For X5, worsening indicates a more negative value; for all other IOS measures, worsening indicates a higher value.

Panel B. Exploratory classification according to the X5 cutoff in patients with non-IPF ILD

X5 category	Progression	No progression	Total
**Abnormal (X5 <−0.085 kPa·s/L)**	28	24	52
**Normal**	0	116	116
**Total**	28	140	168

Metric	Estimate (exact 95% CI)
**Sensitivity**	100.0% (87.7–100.0%)
**Specificity**	82.9% (75.6–88.7%)
**PPV**	53.8% (39.5–67.8%)
**NPV**	100.0% (96.9–100.0%)
**Accuracy**	85.7% (79.5–90.6%)

Notes: The X5 threshold was derived in the full cohort against spirometry-defined SAI and was applied unchanged in the non-IPF subgroup. Exact binomial 95% confidence intervals are reported for classification measures. No progression events were observed among participants with normal X5 in this cohort; this finding should not be interpreted as evidence of zero risk or as a validated clinical rule-out threshold.

Supplementary Table S6. Secondary exploratory cutoff-based associations with progression.

Parameter	Exploratory cutoff	Abnormal n	Adjusted Firth OR (95% CI)	P value
**R5**	>0.402	119	1.77 (0.79–4.00)	0.163
**R20**	>0.307	113	1.06 (0.50–2.28)	0.872
**Mean R5–R20**	>0.364	108	1.14 (0.51–2.50)	0.748
**X5**	<−0.085	76	376.55 (51.40–48013.95)	<0.001
**AX**	>0.675	73	4.60 (2.11–10.31)	<0.001
**Fres**	>17.715	74	1.62 (0.75–3.46)	0.213

Note: Firth penalized logistic regression was adjusted for age, sex, height and baseline FVC % predicted. Cutoffs were internally derived against spirometry-defined SAI and are presented only as secondary exploratory thresholds.

Supplementary Table S7. Exploratory classification performance of IOS cutoffs against spirometry-defined SAI.

Parameter	Cutoff	AUC (95% CI)	Sensitivity (95% CI)	Specificity (95% CI)	PPV (95% CI)	NPV (95% CI)	Accuracy (95% CI)
**R5**	>0.402	0.659 (0.583–0.735)	66.2% (53.7–77.2%)	60.4% (53.0–67.5%)	37.8% (29.1–47.2%)	83.1% (75.7–89.0%)	62.0% (55.7–67.9%)
**R20**	>0.307	0.620 (0.544–0.696)	60.3% (47.7–72.0%)	61.5% (54.1–68.5%)	36.3% (27.4–45.9%)	81.0% (73.6–87.1%)	61.2% (54.9–67.2%)
**Mean R5–R20**	>0.364	0.642 (0.566–0.718)	61.8% (49.2–73.3%)	64.7% (57.4–71.5%)	38.9% (29.7–48.7%)	82.3% (75.2–88.1%)	63.9% (57.7–69.8%)
**X5**	<−0.085	0.676 (0.599–0.753)	50.0% (37.6–62.4%)	77.5% (70.9–83.3%)	44.7% (33.3–56.6%)	81.0% (74.5–86.5%)	70.2% (64.2–75.7%)
**AX**	>0.675	0.693 (0.620–0.766)	51.5% (39.0–63.8%)	79.7% (73.2–85.2%)	47.9% (36.1–60.0%)	81.9% (75.5–87.2%)	72.2% (66.2–77.6%)
**Fres**	>17.715	0.649 (0.572–0.726)	47.1% (34.8–59.6%)	77.5% (70.9–83.3%)	43.2% (31.8–55.3%)	80.1% (73.5–85.7%)	69.4% (63.4–75.0%)

Note: The positive reference category was spirometry-defined SAI. These thresholds were internally derived and are not proposed as clinical diagnostic cutoffs.

Supplementary Table S8. Internal validation and stability of ROC-derived IOS thresholds against spirometry-defined small airway impairment.

Panel A. Bootstrap-based internal validation of continuous ROC AUC

IOS parameter	Apparent AUC	Bootstrap AUC (95% CI)	Mean OOB AUC	.632+ corrected AUC
**R5**	0.659	0.658 (0.582–0.737)	0.662	0.661
**R20**	0.620	0.621 (0.542–0.696)	0.620	0.620
**Mean R5–R20**	0.642	0.642 (0.559–0.721)	0.642	0.642
**X5**	0.676	0.678 (0.600–0.754)	0.676	0.676
**AX**	0.693	0.693 (0.620–0.762)	0.688	0.690
**Fres**	0.649	0.646 (0.573–0.722)	0.649	0.649

Panel B. Repeated cross-validation

IOS parameter	Repeated 10-fold CV AUC, mean ± SD
**R5**	0.664 ± 0.106
**R20**	0.638 ± 0.104
**Mean R5–R20**	0.651 ± 0.094
**X5**	0.682 ± 0.120
**AX**	0.690 ± 0.122
**Fres**	0.659 ± 0.129

Panel C. Stability of internally selected Youden cutoffs

IOS parameter	Apparent Youden cutoff	Bootstrap-derived cutoff 95% interval
**R5**	>0.402	0.310–0.578
**R20**	>0.307	0.233–0.333
**Mean R5–R20**	>0.364	0.266–0.482
**X5**	<−0.085	−0.130 to −0.050
**AX**	>0.675	0.188–1.241
**Fres**	>17.715	11.250–20.120

Notes: Spirometry-defined SAI was used as the positive reference category. Apparent AUCs were calculated from the continuous IOS measurements, with 95% confidence intervals estimated using DeLong's method. Ordinary bootstrap validation used 500 resamples. Within each bootstrap resample, ROC analysis and Youden-index cutoff selection were repeated; continuous IOS measurements were used to evaluate AUC, whereas the bootstrap-selected cutoff was used to assess classification performance in the corresponding out-of-bag observations. The .632+ estimates combined apparent and out-of-bag AUCs using adaptive .632+ weighting. Repeated 10-fold cross-validation was performed with 10 repetitions. Bootstrap cutoff intervals are percentile-based 95% intervals of the re-estimated Youden thresholds. These analyses were secondary and exploratory.

Supplementary Table S9. Baseline demographic characteristics, spirometric indices and impulse oscillometry parameters in healthy controls and patients with interstitial lung disease.

Variable	Healthy controls (n = 51)	ILD patients (n = 255)	P value
**Demographics**			
**Age, years**	55.0 (48.5-61.0)	68.0 (58.0-74.0)	<0.001
**Male sex, n (%)**	31 (60.8%)	135 (52.9%)	0.31
**Body mass index, kg/m²**	24.65±4.48	24.72±3.42	0.90
**Spirometry**			
**FVC, % predicted**	98.0 (90.5-107.5)	89.0 (76.0-100.0)	<0.001
**FEV_1, % predicted**	99.0 (93.5-112.0)	87.0 (75.0-100.0)	<0.001
**FEV_1/FVC, %**	79.0 (78.0-82.0)	78.0 (72.0-82.0)	0.01
**Impulse oscillometry**			
**R5, kPa·s/L**	0.32 (0.29-0.35)	0.39 (0.31-0.51)	<0.001
**R20, kPa·s/L**	0.27 (0.24-0.31)	0.29 (0.23-0.35)	0.08
**Mean R5–R20, kPa·s/L**	0.29 (0.27-0.32)	0.33 (0.27-0.43)	<0.001
**X5, kPa·s/L**	-0.05 (−0.06~-0.04)	-0.06 (−0.09~-0.03)	0.19
**AX, kPa/L**	0.10 (0.06-0.22)	0.40 (0.19-0.77)	<0.001
**Fres, Hz**	9.82 (8.67-12.69)	15.68 (12.58-18.06)	<0.001

Notes: P values are unadjusted. Because healthy controls were substantially younger than patients with ILD and were not age matched, these comparisons are presented only as descriptive physiological reference data and were not used as primary inferential evidence. Device-reported Mean R5–R20 was extracted directly from the IOS system output and should not be interpreted as the arithmetic difference between the separately reported R5 and R20 summary values.

Supplementary Table S10. Actual outcome assessment timing.

Assessment timing	Progressors (n=41)	Non-progressors (n=214)	Overall (n=255)
**Time from baseline, months, median (IQR)**	12.1 (11.4–12.9)	12.0 (11.3–12.8)	12.0 (11.3–12.8)
**Range, months**	9.6–14.7	9.2–14.9	9.2–14.9
**Within 10–14 months, n (%)**	37 (90.2%)	190 (88.8%)	227 (89.0%)
**Within 11–13 months, n (%)**	28 (68.3%)	147 (68.7%)	175 (68.6%)

Supplementary Table S11. Narrower-window sensitivity analysis for continuous IOS associations.

IOS parameter	Primary 9–15 mo adjusted OR	10–14 mo adjusted OR
Fres	1.61 (1.11–2.36)	1.44 (0.95–2.20), P=0.087
R5	1.36 (0.96–1.96)	1.28 (0.86–1.91), P=0.224
R20	1.11 (0.75–1.60)	1.06 (0.69–1.62), P=0.790
Mean R5–R20	1.33 (0.92–1.90)	1.24 (0.82–1.88), P=0.310
X5	8.89 (4.66–19.47)	7.65 (3.72–17.20), P<0.001
AX	1.67 (1.22–2.38)	1.55 (1.07–2.27), P=0.021

Notes: Firth penalized logistic regression adjusted for age, sex, height and baseline FVC % predicted. Odds ratios are expressed per 1-SD worsening using the SD scaling derived from the full cohort.

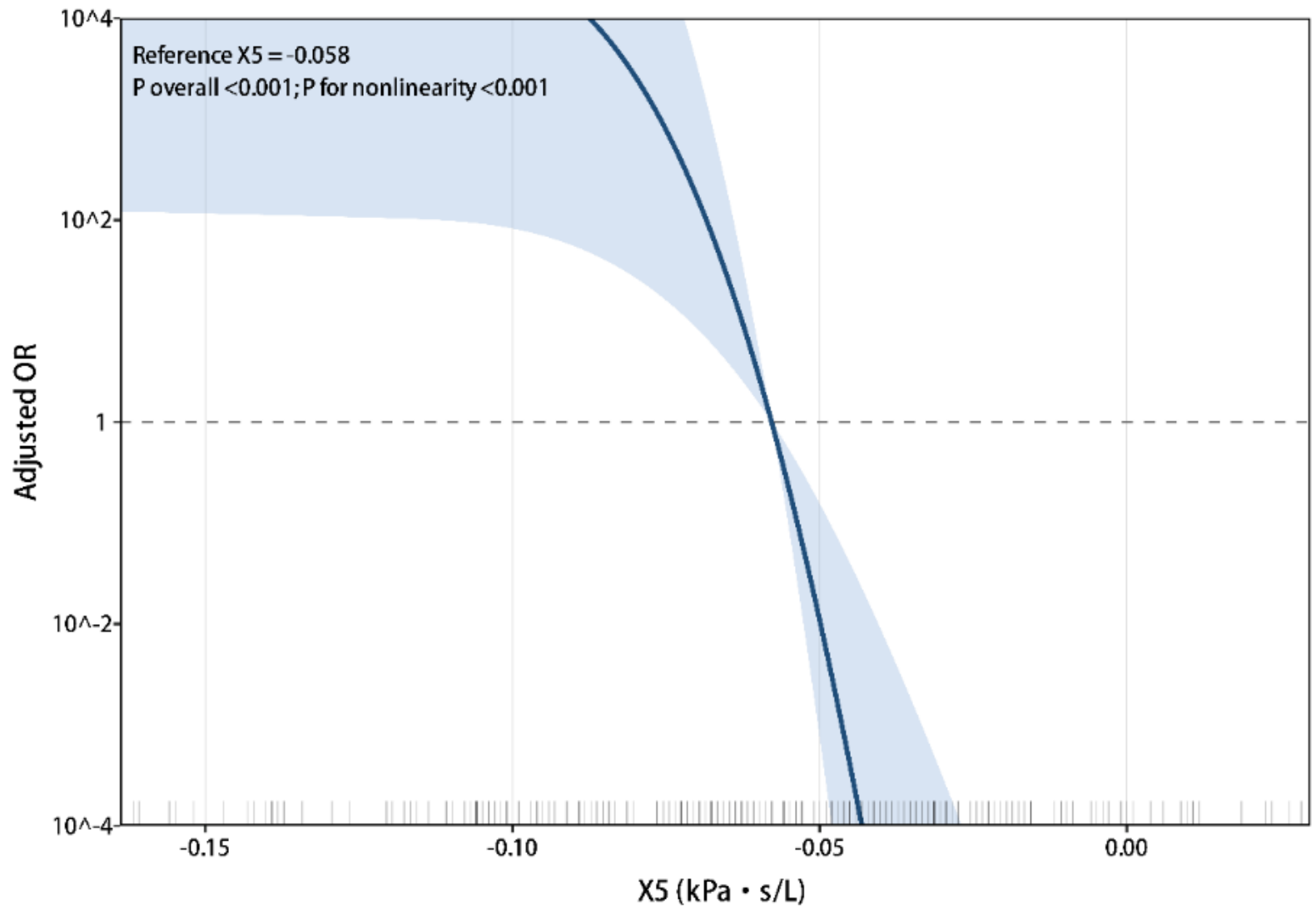

Supplementary Figure S1. Restricted cubic spline analysis of the nonlinear association between X5 and fibrotic disease progression in patients with interstitial lung disease. The solid line represents the adjusted odds ratio and the shaded area represents the 95% confidence interval. The median X5 value was used as the reference (OR=1). The model was adjusted for age, sex, height and baseline FVC % predicted. The spline was fitted using Firth penalized logistic regression adjusted for age, sex, height and baseline FVC % predicted.

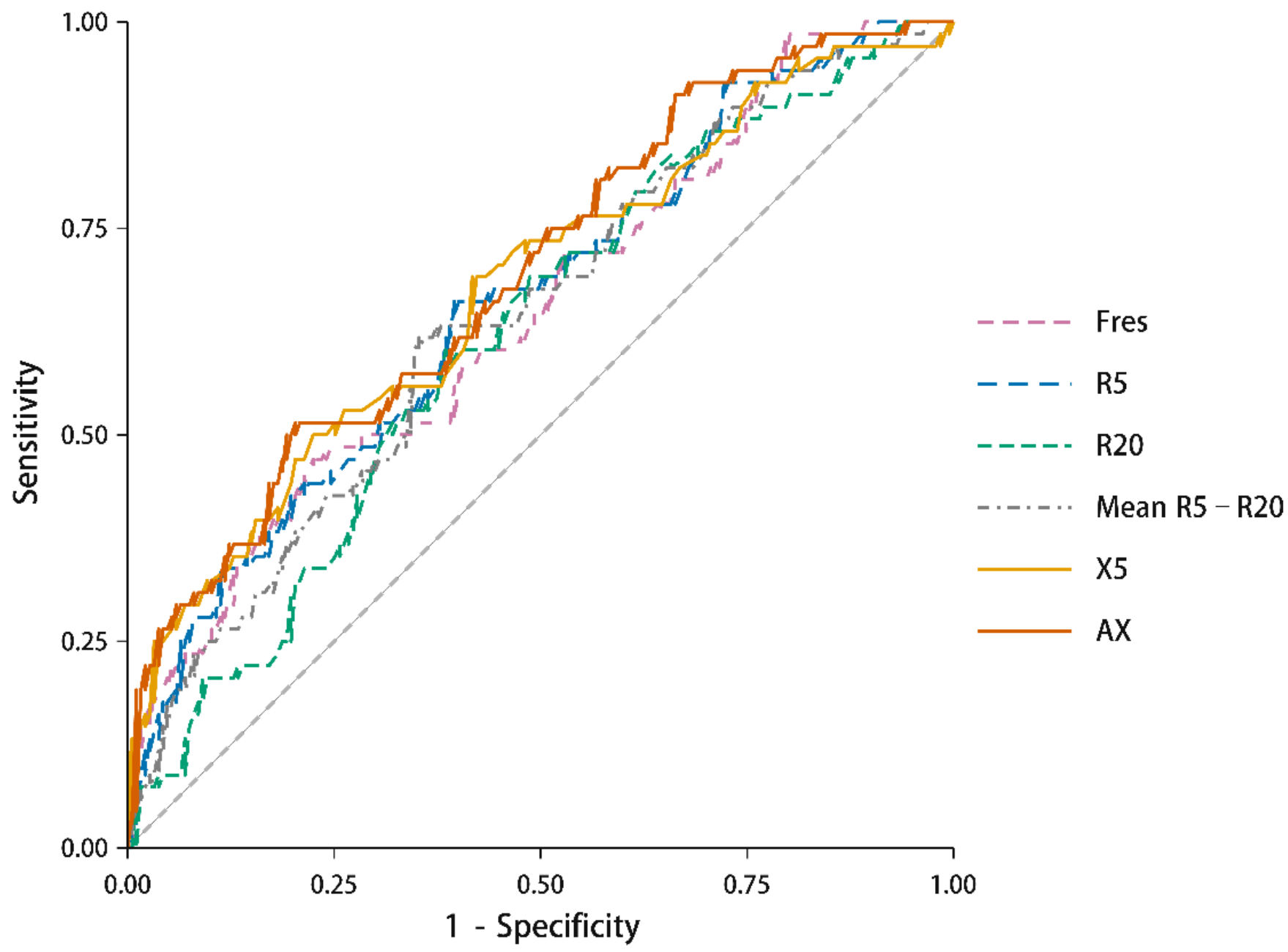

Supplementary Figure S2. Receiver operating characteristic curves showing the discriminative overlap between impulse oscillometry parameters and spirometry-defined small airway impairment. Curves are shown for Fres, R5, R20, device-reported Mean R5–R20, X5 and AX. The diagonal reference line represents an AUC of 0.50.
